# Thromboembolic risks of recombinant factor VIIa use in warfarin-associated intracranial hemorrhage

**DOI:** 10.1186/cc9853

**Published:** 2011-03-11

**Authors:** S Chou, X Cai, R Konigsberg, L Bresette, G Henderson, F Sorond, A Ropper, S Feske

**Affiliations:** 1 Brigham and Women's Hospital, Boston, MA, USA

## Introduction

Recombinant factor VIIa (rFVIIa) may produce rapid hemostasis in warfarin-associated intracerebral hemorrhage (WICH) but may carry high thromboembolic risks. We compared baseline thromboembolic risk factors and thromboembolism rates in WICH patients treated with rFVIIa to those treated with FFP and vitamin K alone.

## Methods

We identified 45 consecutive WICH patients treated with rFVIIa and 34 treated with FFP and vitamin K, and compared their incidence of pre-existing thromboembolic risk factors, troponin elevation, EKG changes, ischemic stroke, pulmonary embolism (PE), and deep vein thromboses (DVT).

## Results

Both rFVIIa-treated and control WICH patients have high prevalence of pre-existing thromboembolic risk factors including atrial fibrillation (73% vs. 68%), DVT/PE (10% vs. 6%), coronary artery disease (CAD) (38% vs. 32%), and abnormal EKG (78% vs. 85%). Troponin elevation is common in WICH and incidence of troponin elevation (47% vs. 41%) and clinically significant myocardial infarction (MI) (13% vs. 6%) are similar between treatment groups. Past history of CAD (*P *= 0.0061) and baseline abnormal EKG (*P *= 0.02) were independently associated with clinically significant MI following WICH. Incidence of DVT/PE (2% vs. 9%) and ischemic stroke (2% vs. 0%) are comparable between rFVIIa-treated and control groups. Recombinant FVIIa-treated patients had lower mean INR at 3 (*P *= 0.0001) and 6 hours (*P *< 0.0001) and received fewer units of FFP transfusion (3 vs. 5; *P *= 0.003).

## Conclusions

Recombinant FVIIa use in WICH is not associated with increased thromboembolic complications compared with FFP and vitamin K alone and may decrease the quantity of FFP use. A prospective randomized study is necessary to determine whether rFVIIa improves outcome of WICH.

